# “I Still Need Your Help”: Online information seeking behavior of International Students in the United States on Reddit

**DOI:** 10.1371/journal.pone.0341314

**Published:** 2026-02-09

**Authors:** Sangpil Youm, Chaeeun Han, Hojeong Yoo, Sou Hyun Jang, Bonnie J. Dorr

**Affiliations:** 1 Department of Computer and Information Science and Engineering, University of Florida, Gainesville, Florida, United States of America; 2 College of Information Sciences and Technology, Penn State University, University Park, Pennsylvania, United States of America; 3 Institute for Health Informatics, University of Minnesota, Minneapolis, Minnesota, United States of America; 4 Department of Sociology, Korea University, Seoul, South Korea; NYU Grossman School of Medicine: New York University School of Medicine, UNITED STATES OF AMERICA

## Abstract

This study examines the online information-seeking behavior of international students in the United States. Following the onset of COVID-19, their need for timely and relevant information becomes critical. Despite greater challenges than domestic students, limited research explores how international students use online platforms to meet their unique information needs. With online communities being essential sources of information and bridges for online social capital, our study analyzes the *r/f1visa* subreddit to examine international students’ information-seeking patterns before and during the COVID-19 pandemic. Additionally, we identify unmet information needs through members’ interactions and recurring questions. Our analysis reveals a shift in topics, with pandemic discussions focusing on travel, financial difficulties, and entry concerns, while pre-pandemic conversations primarily about employment. The increased similarity among recurring questions during the pandemic suggests a convergence of shared struggles that fosters solidarity and emotional support, even as many informational needs remain inadequately addressed. By examining international students’ information needs through the theoretical lens of online social capital, this study contributes to understanding how crisis conditions reshape the dynamics of online communities, blurring traditional distinctions between bonding and bridging capital. The findings can inform universities, policymakers, and online community designers in developing more responsive and inclusive information environments that recognize both the instrumental and emotional support functions of digital platforms for international students.

## Introduction

The number of students who have crossed international borders for educational participation [1–3] increased from 2 million in 2000 to over 6.3 million in 2020 [2]. The United States remains the leading destination for international students, with around one million students from over 200 countries pursuing higher education there as of the 2022–2023 academic year [4]. These students constitute approximately 5.6% of the total college student population [5]. Previous studies have found that international students often experience various difficulties, including academic challenges, language and cultural barriers, social isolation [6], assimilation and identity issues [7], and perceived discrimination [8]. In addition, limited language proficiency and social networks further hinder their ability to access essential information for daily life, such as on legal, financial, and relationship matters and personal development, rather than academic information, in the destination country [9].

College students in recent years face unique challenges compared to older generations, including navigating the transition to the Fourth Industrial Revolution, which is marked by the rise of online learning that demands increased digital literacy. In addition, global issues such as climate change, the Russia–Ukraine War, and widespread anti-war movements contribute to a complex environment for students. These rapid societal shifts, compounded by the sudden global crisis of the COVID-19 pandemic, have had largely negative impacts on college students overall, affecting various aspects such as academic performance, mental health issues, and a sense of connectedness and belonging [1,10–12]. International students are particularly vulnerable during this crisis [13,14], and tend to report high levels of mental health issues [15,16], microaggressions and discrimination targeting Chinese and other Asian international students [13,17–19], unmet mental health needs [1,13], and financial difficulties [20]. Moreover, international students are often required to undergo mandatory quarantine upon arrival in the destination country [21], a measure that restricted mobility for approximately 89% of them during the COVID-19 pandemic [22]. They also express concerns about their future careers and opportunities for transnational mobility [23,24], challenges that domestic students typically do not face.

Despite the increased demand for various types of information during the uncertainty of the COVID-19 pandemic [25], and evidence that active information-seeking enhances college students’ well-being [26], research on international students’ information-seeking behavior remains limited. While Jang and Yi [27] examine health information-seeking among Chinese international students, and other studies explore information-seeking in the general population during the pandemic [28], these studies overlook the broader information needs of international students in natural online settings.

This gap is particularly significant given that international students primarily rely on the internet and social media platforms for information [1,9,29,30]. Beyond understanding what types of COVID-19–related information students seek [31], it is essential to examine how international students interact with each other online and identify their unresolved information needs in order to provide more effective support. Online social capital helps mitigate the loss of social networks that migrants and international students experience when crossing borders [32], and it plays a crucial role in supporting their adaptation. Online communities such as Reddit, where members actively exchange questions, share information, and offer emotional support within topic-specific subreddits, can therefore serve as valuable resources for international students navigating uncertainty during global crises.

Recent studies confirm that Reddit is a suitable platform for examining international students’ information-seeking behaviors. Its predominantly English-language environment, large U.S.-based user community, public accessibility, and user anonymity make it well suited for observing candid discussions within topic-specific communities such as *r/InternationalStudents* and *r/f1visa* [33]. Comparative research further shows that international students actively use Reddit to seek information and manage uncertainty before moving abroad [34]. Therefore, this study uses Reddit to investigate international students’ information-seeking and coping behaviors during the COVID-19 pandemic. In discussions within the subreddit, *r/f1visa* where members engage in “questions and answers about U.S. F-1 visas (academic student visas that allow students to enter the U.S. as full-time students at accredited educational institutions [35])”, this study focuses on the information-seeking behaviors of international students, emphasizing the challenges they face and their unmet information needs.

Studies show that when individuals seeking information in online communities with shared interests encounter unanswered, inaccurate, or outdated responses, their dissatisfaction tends to be significantly high [36–38]. However, there is limited research specifically focusing on the unmet information needs of international students, a critical and distinct group in such online interactions. Since fulfilled information needs serve as indicators of high-quality responses [39], understanding the information needs of international students is essential for providing effective support and informing the design of appropriate solutions [1]. This study, therefore, emphasizes which information needs are fulfilled and which remain unmet; these unmet needs not only highlight gaps in support but also provide valuable insights for improving the design of online communities.

**Fig 1:**
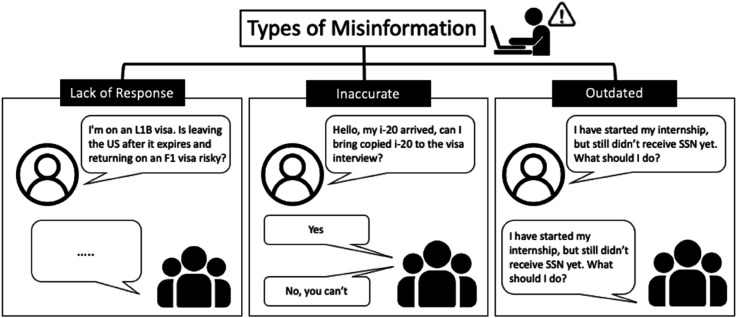
Three types of unmet information needs: lack of responses, inaccurate information, and outdated information.

We identify three types of *unmet information needs*: (i) lack of response [36], (ii) inaccurate information [37], and (iii) outdated content [38], as shown in Fig 1. To the best of our knowledge, this study is the first to categorize unmet information needs specifically within the context of international students. **Lack of response** occurs when a member repeatedly asks the same question but receives no answer. For instance, a member asks about the process of switching from an L1 visa (a non-immigrant visa category in the United States that is designed for intracompany transferees) to an F1 visa, yet no one provides a response. **Inaccurate information** arises when the answers to a member’s question are inconsistent among different respondents. For example, members provide conflicting answers when asked whether a copied I-20 can be brought to a visa interview. Lastly, **outdated information** refers to cases where members are unable to provide timely or relevant updates. An example is when a member asks about the timeline for receiving an SSN; however, no one can provide an answer due to the uncertainty caused by the onset of COVID-19.

This study uses data from the subreddit *r/f1visa*, which includes 8,006 posts and 33,981 comments collected between August 20, 2015, and December 31, 2022. To examine international students’ online information-seeking behavior, it employs the BERTopic model with pre-trained language embeddings and class-based TF-IDF clustering, addressing the following research questions:

- **RQ1:** How do the topics discussed by international students on the *r/f1visa* subreddit shift before and during the COVID-19 pandemic?
- **RQ2:** How does the nature of unmet information needs, reflected by recurring questions, evolve before and during the COVID-19 pandemic, and how do international students on Reddit adapt their communication to address these evolving needs?

These questions investigate how global crises reshape international students’ access to and reliance on online information. RQ1 highlights shifts in their concerns across different periods, offering insights into the broader academic, social, and personal challenges international students face in times of uncertainty. RQ2 extends this analysis by identifying unmet information needs and the communication strategies students adopt in response, thereby revealing structural gaps in support systems.

This study advances three key contributions. First, it demonstrates how online communities reflect the evolving concerns of international students, particularly during crises, thereby advancing research on digital information-seeking behaviors. Second, it identifies recurring unmet information needs and surrounding communication patterns, revealing systemic gaps in existing institutional and policy-level support. Finally, the study’s findings carry theoretical contributions and practical and societal implications, underscoring the need for adaptive, context-sensitive strategies that enhance international students’ resilience, promote equitable access to information, and strengthen the role of online communities as sources of social capital and support.

## Literature review

### Theoretical framework of information-seeking behavior

Wilson’s model of information behavior conceptualizes information seeking as the totality of human actions related to sources and channels of information [40]. In this framework, information need is a secondary-order need that arises from underlying cognitive or affective states of stress or uncertainty [40]. Information-seeking behavior functions as a coping mechanism to reduce uncertainty [40,41] and is shaped by intervening variables such as psychological, social, and environmental factors that either facilitate or constrain access to information [40]. This study extends Wilson’s framework into a computational context, illustrating how international students manage uncertainty and stress through online communities such as Reddit and how information-seeking behaviors operate within digital peer networks during crises like the COVID-19 pandemic. Their posts reflect coping behaviors within a “context of information need” [40], often triggered by challenges related to social integration, housing, healthcare, and administrative systems.

Building on Wilson’s conceptualization, Information Foraging Theory (IFT) provides a complementary perspective that explains how individuals allocate attention and effort once an information need is triggered [42]. IFT models information-seeking as a dynamic optimization process in which individuals aim to maximize informational gain relative to cognitive and temporal costs. Drawing on ecological metaphors, Pirolli and Card describe information environments as “information patches,” evaluated based on perceived “information scent,” or cues indicating potential value or relevance. Decisions to continue exploiting a known patch or explore new ones depend on this cost–benefit calculation.

In online environments such as Reddit, these insights help explain international students’ information-seeking behaviors [42,43]. Once an information need, such as clarifying visa procedures or understanding employment eligibility emerges, users weigh the utility of continued engagement with familiar sources against the potential benefits of seeking new information elsewhere [42,44]. Repeated posts on similar topics, paraphrased questions, and follow-up comments illustrate *re-exploitation* strategies, in which users revisit previously explored patches to obtain updated or more accurate information as conditions evolve [42]. This behavior becomes particularly salient during the COVID-19 pandemic, when rapid policy changes and procedural uncertainties increase the cost of exploration and heighten the value of returning to familiar, trusted sources [45].

To guide this study, we integrate Wilson’s model and IFT, which together illuminate not only why international students seek information online, rooted in the need to reduce uncertainty and overcome structural barriers, but also how they adapt their information-seeking strategies in dynamic environments. This integrated framework informs our analysis of recurring information needs and behavioral patterns, offering a nuanced understanding of how online peer communities operate as essential information infrastructures during periods of uncertainty.

### Information needs and barriers among International Students

Earlier investigations show little variation in information proficiency between domestic and international students [46,47]. However, despite comparable levels of proficiency, international students exhibit distinct information needs and face multiple barriers in their information-seeking processes compared to native-born students [27,39,48–51]. Language and cultural differences often act as barriers to international students’ information literacy and information-seeking behaviors [51,52]. In addition, international students frequently face difficulties when using libraries as information sources due to an unfamiliar technological environment, language and cultural hurdles, and negative perceptions, including feeling overwhelmed [48]. Furthermore, international students struggle to evaluate information credibility and the reliability of sources [9,51], which can compromise the safety and effectiveness of their information-seeking behavior.

Although international students typically rely on the internet as their primary information source, similar to domestic students [9,46,53], they encounter greater obstacles than their domestic counterparts in evaluating information quality, timeliness, quantity, and source appropriateness [50]. Compared to academic information, international students often face more difficulties in locating everyday information, including legal, financial, and personal development resources [9]. Their transition to a foreign environment may involve challenges related to social integration, housing, healthcare, and legal issues, all of which contribute to their distinct needs. In this study, we categorize the topics of challenges international students face and highlight how these topics shifted during the COVID-19 pandemic, thereby systematically examining their information needs.

### Online social capital

With the development of the internet and social networking services, traditional forms of social capital, once grounded in physical interaction, have increasingly shifted into the online realm [54]. Similar to conventional social capital [55], online social capital exists in different forms. On the one hand, online bonding social capital refers to strong, close-knit relationships among homogeneous groups maintained through digital platforms. These connections are typically characterized by emotional support and solidarity [54]. Previous studies show that online ethnic communities, where members share common ethnicity and language, serve as a form of online bonding social capital. These communities provide members with everyday information, transnational connections to their home countries, and emotional comfort [56–58].

The significance of online bonding social capital becomes especially prominent during the COVID-19 pandemic, as members of these communities share not only information about health and safety but also personal experiences, emotional struggles, and coping strategies. In this context, online bonding social capital provides essential emotional and practical support, particularly for individuals experiencing isolation or disconnection from traditional in-person networks, especially within immigrant communities [57].

On the other hand, online bridging social capital plays a crucial role in fostering “strong bridges” among “disconnected strangers” [59], including international students, by extending their networks beyond close and familiar ties. Characterized by connections based on weak ties, this form of capital exposes individuals to diverse perspectives and experiences [55]. Through such interactions, international students broaden their worldviews and develop a deeper understanding of the global community.

Participation in online communities further enhances these effects by providing access to a broad range of information, resources, and opportunities for professional growth, including career advice, job postings, and skill-building activities [60,61]. Moreover, online communities facilitate interpersonal connections that may not arise through traditional in-person networks, helping international students bridge knowledge gaps, foster social integration, and enhance both personal and professional development [62]. Consistent with these dynamics, several studies show that frequent use of social networking sites strengthens bridging social capital among international students by expanding weak ties and increasing the number of acquaintances within their networks [62–64].

However, limited understanding remains regarding how international students engage in online information-seeking behavior within the context of online social capital. Platforms such as the *r/f1visa* subreddit, which foster online social capital, offer a distinctive environment for these interactions. This study applies the framework of bridging social capital to examine how such digital spaces facilitate connections among individuals from diverse backgrounds and promote the exchange of varied perspectives and experiences.

### Computational approach in online information-seeking behaviors

Social media platforms have become indispensable tools for seeking, sharing, and disseminating information [65,66]. This trend is particularly evident among younger generations, especially international students navigating major life transitions [46]. For these students, social media not only sustains connections with friends and family but also serves as a key resource for managing challenges [67].

Compared to other online platforms, such as microblogging sites (e.g., Twitter, now X), wikis, forums, and personal blogs are often perceived as more reliable sources of information because members can access up-to-date content without disclosing personal details [39,65,68]. Given the trust users place in these platforms, their value extends beyond general information sharing to more specialized purposes such as question-and-answer (Q&A) exchanges. Frens et al. [39] define high-quality answers on Q&A platforms as those that meet users’ needs and provide sufficiently detailed information. Such platforms hold considerable potential to facilitate informal, self-directed learning by encouraging the production of high-quality responses.

Building on this understanding, we use Reddit as a large-scale data source to computationally examine online information-seeking behaviors [69]. Reddit’s member-driven structure, frequent updates, and timely responses make it particularly well suited for studying dynamic, real-world information exchange. Its subreddits, topic-specific forums, enable users to co-construct knowledge and engage in collaborative learning through open discussions on shared concerns [70]. These rich, text-based interactions generate extensive, naturally occurring data that support computational analyses of behavioral patterns, discourse topics, and evolving information needs.

While prior research qualitatively examines social media as a tool for information seeking among international students [71,72], few studies systematically analyze the types of information these students seek or the nature of their interactions. Recent computational studies apply content analysis and topic modeling to Reddit data to uncover prevalent themes among users [73–75], yet such methods are rarely applied in the context of international students. To address this gap, this study employs computational methodologies to identify, categorize, and interpret unmet information needs as expressed in Reddit discussions.

Yet, even with large-scale computational analysis, cultural considerations remain essential for accurate interpretation. Prior research emphasizes that effective online community design for minority groups must account for cultural and contextual differences [76,77]. Building on these insights, this study focuses on international students, a group that often faces limited resources and support [78], challenges that have intensified during the COVID-19 pandemic [13,14]. Despite their growing presence in higher education, the specific information needs and online behaviors of international students remain underexplored. This study, therefore, investigates how these needs are articulated and addressed within Reddit’s Q&A environment.

## Data

We use posts and comments from the *r/f1visa* subreddit, which ranks among the top 4% all subreddits by size and includes approximately 30,000 members. Members of this subreddit discuss topics related to the F1 visa, which is the required visa for international students pursuing academic studies in the U.S. [79]. The following section provides an overview of the dataset and describes the procedures used for data collection and preprocessing. All code and data are available in our repository (https://github.com/chaeeun-h/international_student_information_seeking).

### Data collection

In this study, we use the social media platform, Reddit, which has approximately 430 million monthly active members as of 2020 and more than 138,000 active subreddits where people engage in discussions on specific topics [80]. Reddit members can seek information and share content on a variety of topics [81]. This online community becomes one of the most widely utilized information sources during the COVID-19 pandemic, especially for discussions related to public health and social issues [82]. For example, studies have analyzed themes and sentiments on subreddits like *r/coronavirus* and found that topics on Reddit during the pandemic varied according to political ideologies, impacting the credibility of shared information [83,84]. Thus, Reddit remains one of the most widely utilized information sources during the COVID-19 pandemic.

Subreddits are organized around shared topics and interests, which foster bonding and bridging social capital by connecting users with both similar and diverse backgrounds. For international students, these subreddits provide semi-anonymous yet supportive spaces to seek advice, share experiences, and access peer-generated information. Unlike Facebook, which relies heavily on pre-existing social networks, or Quora, which prioritizes expert-driven responses, subreddits encourage community-based knowledge exchange and collective problem-solving that more closely reflect the ways international students build and leverage social capital in their everyday lives.

Given the broad reach and active participation on Reddit, we specifically focus on the subreddit *r/f1visa*, established in 2014, with 30,000 members as of March 2024. This subreddit serves as a communication platform for seeking information related to F1 visa status and is ranked in the top 4% among approximately 140,000 subreddit communities [80]. Another relevant subreddit, *r/InternationalStudents*, includes discussions among international students from various countries, providing a diverse platform for shared experiences and advice. However, we choose not to include it in our analysis because it encompasses students residing in numerous countries beyond the U.S., which dilutes our focus on a specific national context. Scholars have emphasized the importance of the “context of reception,” a concept describing how conditions in the host country—such as perceived welcomeness, available opportunities, racial and ethnic composition, and the presence of social support networks—shape the experiences of international migrants [85]. This context plays a crucial role in influencing international students’ adjustment processes, determining how effectively they adapt to academic, social, and cultural environments in their destination country [86]. By focusing exclusively on international students in the United States, this study aims to capture a more nuanced understanding of how the sociocultural and institutional characteristics of the U.S. context shape their adaptation processes.

**Terms and condition.** The dataset is collected using the Pushshift API on Reddit (https://www.reddit.com/). All data collection, processing, and analysis procedures comply with Reddit’s API Terms of Service and ethical guidelines for research using public data. Only publicly available content shared in accordance with platform policies is utilized. As the data are publicly accessible, Institutional Review Board (IRB) approval and a waiver of consent are not required [87]. To protect user privacy, all identifiable information is removed during preprocessing. The final aggregated dataset contains no personally identifiable information (PII) and cannot be traced back to individual users.

### Data preprocessing

According to the subreddit rules, off-topic posts and comments are removed, and titles without a question or with generic wording such as “I-20 Problem” are also deleted. Consequently, this subreddit serves as a valuable resource for both quantitative and qualitative analyses of collective information through its submissions and comments [88]. Within the *r/f1visa* subreddit, 8,006 original submissions (hereafter referred to as posts) and 33,981 corresponding comments are collected from August 20, 2015, to December 31, 2022. These posts are authored by 3,814 members, while 5,197 members participated through comments.

To examine the role of disclosure in shaping member perceptions before and during the COVID-19 pandemic, we divide the dataset based on the onset of COVID-19 on January 1, 2020. Table 1 presents the dataset statistics after preprocessing. The pre-COVID-19 period comprises 534 posts and 1,325 comments, with an average of 2.48 comments per post. During this time, 325 members publish posts, and 326 contribute to comments. In contrast, the during-COVID-19 period (January 1, 2020–December 31, 2022) includes 7,472 posts and 32,656 comments, with an average of 4.37 comments per post. A total of 3,519 users create posts, and 4,941 participate in comment discussions. The sharp rise in post volume during the pandemic mirrors a broader increase in Reddit activity: from roughly 150 million annual posts in 2018–2019 to over 400 million in 2020, with continued growth thereafter [80].

**Table 1:** *r/f1visa* data overview.

Data Type	All Period	Before COVID-19	During COVID-19
	2015/08/20-2022/12/31	2015/08/20-2019/12/31	2020/01/01-2022/12/31
Number of posts	8,006	534	7,472
Number of comments	33,981	1,325	32,656
Comments per post	4.24	2.48	4.37
Number of post authors	3,814	325	3,519
Number of comment authors	5,197	326	4,941

Before analysis, further preprocessing is applied. For posts, we retain only the following attributes: author, date, title, and main content (body), with the latter referring to the post’s textual content. Posts without identifiable authors (blank, deleted, or removed) are excluded. We then merge the title and body fields, including posts containing either one or both; those missing both are discarded. Temporal divisions into pre- and during-COVID periods are determined using the date of the original post. Additional filtering is conducted to remove off-topic or low-quality content. Posts whose titles or bodies are labeled “deleted,” “removed,” or consisted solely of URLs or repetitive promotional phrases are excluded. We also identify and remove spam or advertisement posts by detecting repeated external links and marketing-related keywords.

For comments, we select the author, date, and body fields. Comments with a blank, deleted, or removed author are excluded regardless of whether the comment body remained available. Tables 2 and 3 provide examples of anonymized and rephrased posts and comments to ensure no personally identifiable information is disclosed.

**Table 2:** Post dataset examples.

Author	Date	Title	Body
Member 1	2015-06-01	Canadian pursuing education in the United States	Hello! Does anyone have any advice for the interview at the embassy or with Homeland Security?
Member 2	2022-12-31	Possibility of F1 visa rejection due to a gap	Hey everyone, I have a two-year gap in my employment history because I was laid off.

**Table 3:** Comment dataset examples.

Author	Date	Body
Member 3	2015-05-01 19:01:01	If Wellington is New Zealand, the positive news is you do NOT need to submit an application.
Member 4	2021-01-31 20:40:40	Your payment should remain valid as long as you retain the same SEVIS number.

Finally, for topic modeling, we add “f1” and “visa” to the stop-word list, given their frequent appearance in this subreddit. Treating these terms as stop words helps eliminate repetitive, context-specific noise and allows the model to focus on more meaningful and distinctive discussion topics.

## Methods

In this study, we implement a computational approach, using topic modeling techniques with the BERTopic model, to uncover the predominant themes within *r/f1visa*. Additionally, we identify recurring questions and assess the consistency of topics addressed by members, ultimately providing insights into the unresolved issues and information-seeking behaviors on the platform.

### RQ1: Identifying topics

**Topic modeling using BERT.** Topic identification offers scalable insight into international students’ perceptions, clarifying what matters most and spotlighting key areas that demand focused analysis [89]. To identify topics, we use the BERTopic model for topic modeling [90], a self-supervised learning language model that represents text input as a sequence of vectors. This model leverages its advanced approach to understand the overarching themes within posts and comments on *r/f1visa*. It first embeds input text documents, such as posts or comments, using a pre-trained language model. Specifically, we input the combined title and body text for each post into the BERTopic model to extract embedded vectors using Sentence-BERT (SBERT), which captures the nuanced semantics of textual content in an embedding space. To facilitate efficient clustering of the embedded text into groups, these embeddings undergo dimensionality reduction using UMAP, with the default parameter settings. These settings ensure that similar posts are placed closer together in the reduced-dimensional space, maintaining semantic relationship without compromising data integrity.

The BERTopic model provides an innovative method for organizing and grouping documents by refining the traditional TF-IDF method. Instead of simply counting word frequency, it focuses on the importance of words within specific topic groups. This approach allows the model to better capture the essence of each group, making it more effective than older models. To be specific, it pays attention to the unique words that define each cluster, ensuring that the main themes are clearly identified.

Despite these strengths, BERTopic’s representations are still generated from bag-of-words. Consequently, the listed words do not fully capture the true meaning of the topic, as they only approximate its importance and may appear redundant. To associate identified topics with individual posts, each post is automatically assigned to a topic cluster based on its embedding and proximity to the centroids of the clusters formed by UMAP. However, the final labels for these clusters are not generated automatically; they are manually curated. Using the keywords representing each topic provided by BERTopic, we manually refine the cluster names by leveraging a Large Language Model (ChatGPT) and human consensus, as described in later this section.

Additionally, when used for analyzing social media customer reviews, BERTopic has been found to be more efficient and coherent than other popular techniques, requiring minimal data preparation while still delivering strong results [91]. This means that BERTopic not only groups similar documents but also does so in a way that makes the underlying themes more visible, helping us better understand the key issues being discussed in the data.

By choosing BERTopic for our analysis of *r/f1visa*, we aim to uncover the predominant themes within the community’s discourse, particularly in the context of international students navigating visa issues amid the COVID-19 pandemic. This approach enables us to systematically analyze the platform’s content, revealing insights into the concerns, questions, and information needs that characterize interactions among community members.

**Coherence score for selecting optimal number of topics.** To determine the optimal number of topics, we measure the coherence score, which assesses how well a topic is supported by a set of texts called the reference corpus. This score is based on statistical analysis and probabilities from the reference corpus, focusing on word context.

We begin by selecting a topic and reference corpus, identifying the topic’s top-*n* most significant words (*w*), and employing a segmentation technique to divide *w* into subset pairs. We then calculate the probabilities for these segments using a technique (*P*) based on the reference corpus and assess the relationship between subset pairs within each segment. These assessments were combined into a single numerical value, the topic coherence score, which ranged from 0 to 1; higher scores indicate better coherence.

Specifically, we compare each topic against all others using the *N* most probable words to create a word vector for each topic. Each vector element represents Normalized Pointwise Mutual Information (NPMI) between a focal word and another word in a set. By aggregating these vectors into a topic vector, we derive a coherence score, thereby streamlining the process of evaluating topic coherence.

With the topics extract using the BERTopic model, the Gensim package is employed to assess the optimal number of topics (*N*) by comparing the coherence scores resulting from different values of *N* within the set 4, 5, 6, 7, 8, 9. Following the evaluation, *N* is determined to be 6 (coherence score = 0.8575), as shown in Table 4. Subsequently, each Reddit post is assigned to one of these six topics, thereby ensuring the effective labeling of posts into coherent topic groups.

**Table 4:** Coherence scores per number of topics.

Number of Topics	Coherence Score
4	0.8338
5	0.8448
6	**0.8575**
7	0.7154
8	0.7936
9	0.7878

**Assigning topic titles using a large language model.** As a result of topic modeling, the model generates representative keywords for each topic. We use ChatGPT, a Large Language Model (LLM), to define these topics. Although ChatGPT serves as a valuable resource for describing and identifying topics [92], it occasionally produces information that may not be fully relevant to the given topic. To address this issue and enhance performance, human oversight is necessary to provide more accurate descriptions of topics [93].

Building on prior research, which demonstrates the effectiveness of LLMs in generating appropriate topic names [94], we adopt a specific strategy tailored to this study. We exclude approaches that require inputting the entire document or all documents, as these are impractical for handling large datasets. Instead, we follow a method that merges the keywords representing topic groups extracted by BERTopic model to generate a complete topic name.

To refine this process, we implement a new prompt strategy where the LLM generates three candidate topic names based on the merged keywords using the following prompt:

*“Please provide three topic names based on the words: XYZ.”*

These three options are then evaluated through human interpretation, with our research team reaching a consensus on the final topic name for each topic. This combined approach leverages the efficiency of LLM-generated suggestions while ensuring the accuracy and relevance of the final topic names through human oversight.

### RQ2: Identifying recurring questions among Reddit posts

**Unmet information needs.** Cosine similarity is a method used to measure the similarity between two vectors, ranging from -1 to 1. A value near -1 indicates strong dissimilarity, 0 signifies no relation, and a value close to 1 suggests high similarity [95]. In this study, we apply this method to assess how consistently an author addresses similar topics in their Reddit posts.

Each Reddit post is represented as an embedding vector generated using BERTopic, which captures the semantic content from both titles and body text. For two posts as *A* and *B*, their cosine similarity, calculated using the following equation, indicates how closely related their meanings are:


$$\cos\theta =\frac{𝐀\cdot 𝐁}{\left\|𝐀\|\|𝐁\right\|}=\frac{\sum_{i=1}^{n}A_{i}B_{i}}{\sqrt{\sum_{i=1}^{n}A_{i}^{2}}\sqrt{\sum_{i=1}^{n}B_{i}^{2}}}$$

To determine whether two posts reflect a recurring question, we analyze the empirical distribution of cosine similarity scores across all post pairs written by the same author. Posts on unrelated topics (e.g., travel vs. tax) score below 0.13, while those addressing a similar issue (e.g., visa interview) show similarity values above 0.34. Based on this observation, we set 0.34 as a threshold separating semantically related from dissimilar/unrelated content.

Pairs of posts with cosine similarity above 0.34 are labeled as recurring questions, indicating that the author revisits similar topics or unresolved issues across posts. This approach systematically captures patterns of repeated inquiry while minimizing noise from marginally related content, providing insights into users’ persistent information needs and challenges.

**Investigating comments.** We analyze self-comments made by authors on their posts, with a particular focus on the last comment they posted. This behavior is selected because it offers a direct lens into the authors’ satisfaction or dissatisfaction with the responses they received [96]. Unlike analyzing comments from other users, which can involve information that needs fact-checking, examining self-comments provides clearer insight into whether the information-seeking process fulfilled the authors’ needs.

Our primary interest lies in the behavior of the question-asker—specifically, whether they revisit their posts to follow up with additional questions or conclude their discussions by expressing gratitude with phrases such as “Thank you.” These patterns indicate the responses are perceived as satisfactory. By studying these self-comments, we aim to determine how effectively the information needs of different user groups are addressed in online environments.

This approach aligns with prior research emphasizing the importance of user engagement and satisfaction in evaluating the success of information-seeking behavior. Rooted in frameworks such as Uses and Gratifications Theory, which emphasizes that individuals actively seek to fulfill specific needs online, our analysis highlights how self-commenting serves as both a measure of user satisfaction and an indicator of successful engagement in the information-seeking process [97].

Through this analysis, we uncover valuable insights into the outcomes of information-seeking efforts, assess the success rate of these processes, and identify potential gaps or challenges faced by users in fulfilling their informational needs.

## Results

Using computational methods, we identify the topics that community members seek and how they evolve with the onset of COVID-19. We then uncover frequently or repeatedly asked questions during the pandemic and observe a tendency for members who ask more questions to revisit their posts and leave comments seeking additional information. We determine whether these information inquiries are resolved by analyzing linguistic cues in their follow-up comments.

### RQ1: Topics related to the information needs of International Students

Tables 5 and 6 compare emerging topics among members who post two or more times in both periods, highlighting changes in topics over time. To protect privacy, all examples in these tables have been anonymized and paraphrased to remove any personal identifiers. Before the COVID-19 pandemic, six distinct themes predominantly focus on employment prospects and visa-related queries. These include topics such as the processing of United States Citizenship and Immigration Services (USCIS) Employment Authorization Document (EAD) applications, job opportunities for data scientists and analysts, tips for navigating visa interviews, available web developer positions, requirements for Curricular Practical Training (CPT) jobs, and entry-level job opportunities for attorneys and analysts in the U.S. Specifically, the extended processing times and bureaucratic hurdles associated with the USCIS EAD application process are related to individuals awaiting a new EAD card, demonstrating procedural delays and their direct effect on legal work authorization. Similarly, the quest for entry-level data scientists and analysts indicates challenges related to sponsorship requirements and a high rejection rate, despite customized job applications, highlighting the obstacles to employment posed by visa status limitations.

**Table 5:** Topics among posts of individuals who posted two or more times before the COVID-19 pandemic.

	Topic	Keywords	Representation Docs	%
1	USCIS EAD Application: Processing Time, Card Receipt, and Start Date	EAD, Applications, Card	I’m currently waiting on my new EAD card. Notice says my current card is extended 180 days, but my license tied to it expires this month. Tried renewing at the DMV, but they said the 180-day notice is not enough. Does anybody have any advice?	28
2	Data Scientist and Analyst Jobs: Entry-level Openings in the USA	Data, Jobs, Scientist, Data Analyst	I applied to various roles in data-related fields, customized resume for 300 job applications with cover letters. I received 18 calls, but 12 were rejected due to sponsorship needs. Despite good interviews, faced rejections, feeling frustrated with lack of support from companies.	28
3	Navigating B1/B2 Visa Interviews: Passport Requirements and Waiver Information	Interview, B1, Passport, Waiver, B2	On a B1 visa, I married a US green card holder. My parents-in-law offered to sponsor my legal education. [...] Despite delays in my visa extension and change of status application, I am aiming to switch to F1 status for the spring semester. What are my chances of changing from B1 to an F1 visa successfully?	18
4	Web Developer Jobs: Entry-level Opportunities in the USA	Developer, Entry Level, Jobs, Web	I graduated in June and have been studying web developer. [...] Proficient in HTML, CSS, JavaScript, and Python, I wonder if these skills are enough for entry-level developer and if there are specific qualifications needed.	15
5	CPT Jobs in the USA: Requirements and Search Strategies	CPT, Jobs, Requirements, USA	My OPT ends Feb 11, 2019. Current company wants me to stay but I prefer not to. If my MS Finance degree gets STEM designation approved, can I apply for STEM extension even if I leave the company after the contract?	7
6	Entry-level Attorney and Analyst Jobs: Opportunities in the USA	Entry Level, Attorney, Jobs, Opportunities	Discover entry-level attorney job openings in New York, USA, that enhance career prospects.	4

**Table 6:** Topics among posts of individuals who posted two or more times during the COVID-19 pandemic.

	Topic	Keywords	Representation Docs	%
1	Mastering the Financial Interview: Providing Proof of Funds of University Admission	Interview, Bank, Funds, Loan, Financial	I am using a loan sanction letter from a private bank for faster I20 processing, planning to switch to a loan from a public bank for the visa interview. Is this acceptable to have different loan sources in the I20 and visa interview?	26
2	Optimizing STEM Internships: A Guide to CPT and Working Hours	CPT, Time, Company, Internship, STEM, Degree, Major	Due to an oversight in my timesheet, I worked 22 hours one week without realizing it, and it was approved. Should I be worried?	20
3	Preparing for a New Academic Year: Essential Travel Tips for International Students	New, Travel, Country, Home, Classes, SEVIS, Year	Due to Covid, I haven’t visited my home country since arriving in the US in January 2021. My family advises against travel due to Covid concerns. Will this affect my F1 visa renewal for my master’s program?	18
4	Crucial Steps in EAD Application: A Comprehensive Guide for STEM Students	EAD, USCIS, Date, DSO, Application, Card	Following USCIS’s announcement on Covid flexibilities, OPT applications now receive full recommended OPT time. My program ended on May 2nd, 2020, but my EAD is valid until July 2nd, 2021. I seek a correction to extend it to August 20th, 2021 to recover lost authorization, but I am concerned about remaining EAD time and STEM-OPT risks.	14
5	Navigating IRS Requirements: Essential Tips for Filling Your Taxes	Tax, Taxes, Resident, IRS, Insurance, Non-Resident	As an international student considered a non-resident for tax purposes, I worked for my school’s employer and earned gains from stock trading. Can someone clarify which forms I need to file this year and how to pay taxes on capital gains to the IRS?	13
6	Common Reasons for Passport Refusal and How to Address them	Interview, Embassy, Administrative, Processing, Passport, Documents	Has anyone successfully obtained an F-1 visa after being subjected to administrative processing during the US government shutdown? If so, what was the duration of the process? Are there any steps I can take to expedite the process or receive an estimated timeframe?	9

However, following the onset of COVID-19, the focus shifts towards adapting to the new circumstances caused by the pandemic. Although topics during the COVID-19 are broadly related to employment, they indicate new and various specific challenges faced by international students, such as financial interviews, academic planning, and tax-related concerns. As Table 6 shows, six different topics emerge: mastering financial interviews for university admission, optimizing STEM internships with guidance on CPT and working hours, making preparations for a new academic year with essential travel tips for international students, handling crucial steps in the EAD application processes for STEM students, navigating Internal Revenue Service (IRS) requirements for filing taxes, and discussing common reasons for passport refusal and strategies to address them.

Specifically, mastering financial interviews reflects concerns about using different loan sources for I-20 and visa interviews, indicating the complexities of funding education in uncertain financial times. The topic of optimizing STEM internships addresses the challenges of adhering to CPT requirements in a remote work environment. For example, an individual accidentally exceeding work hours showcases the new logistical and regulatory hurdles students face. The crucial steps in the EAD application process during-COVID-19 mirror pre-pandemic concerns but are compounded by additional layers of complexity due to COVID-19 flexibilities, such as extensions and adjustments to Optional Practical Training (OPT) time. This continuity and evolution of concerns from the pre- to during-pandemic periods reflect ongoing anxiety over employment authorization amid changing immigration policies. Furthermore, the emergence of new topics, such as preparing for a new academic year with essential travel tips for international students, highlights the added dimension of travel restrictions and visa uncertainties affecting academic planning and family reunions. This shift highlights a broadening of information needs from procedural hurdles to more existential challenges that affect students’ personal lives and academic continuity.

### RQ2: Recurring unmet information needs

**Fig 2:**
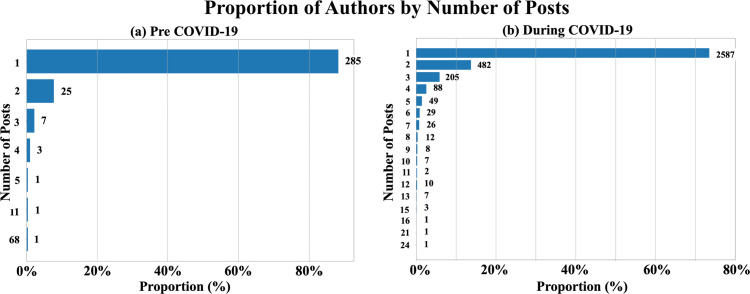
Number of questions before and during the COVID-19 pandemic. (a) shows the ratio of authors who posted a specific number of times compared to the total number of authors before COVID-19. (b) presents the same during COVID-19.

As Fig 2 illustrates, most members post only one question both before and during the COVID-19 pandemic. Specifically 88% of members (285 out of 325 authors post only once before COVID-19, where 73% of members (2587 out of 3519 authors) post during COVID-19. Meanwhile the proportion of members posting more than one question has increased from 12% (38 authors) before COVID-19 to 27% (933 authors) during COVID-19. This includes a notable rise in members posting two questions or the three questions. The increased engagement among these members during the pandemic suggests significant shifts in their behavior and emphasizes the importance of exploring their motivations and posting patterns in more detail.

**Fig 3:**
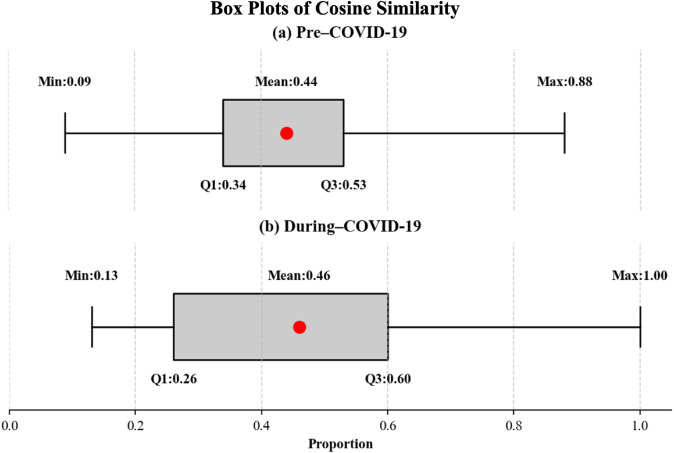
Box plots of cosine similarity. (a) Plot describing cosine similarity among the posts of individual authors who post more than two posts *BEFORE* the COVID-19. (b) Plot describing cosine similarity among posts of individual authors who posted more than two posts *DURING* the COVID-19.

As shown in Fig 3, members who post multiple times during both periods exhibit non-negative cosine similarity among their posts, indicating semantic closeness and a tendency to ask recurring questions on similar topics. Although members address similar topics in their recurring questions during both periods, the topics during the COVID-19 period become more focused, as evidenced by an increase in cosine similarity. Comparing the cosine similarity of topics before the COVID-19 pandemic (0.09, 0.44, and 0.88 for minimum, mean, and maximum, respectively) with that during the pandemic (0.13, 0.46, and 1.0) reveals a slight increase in similarity during the pandemic. This suggests that recurring questions during COVID-19 period are more concentrated on similar topics.

**Types of unmet information needs.** In addition to assessing the information sought before and during the COVID-19 pandemic, we explore the unaddressed information needs identified through an analysis of recurring inquiries among international students. First, we identify three types of unmet information needs: lack of responses, inaccurate information, and outdated content. These issues highlight the challenges international students face as they repeatedly seek necessary information on Reddit.

A common issue is the frequent lack of responses. Among all the posts submitted on the *r/f1visa* subreddit, 20% of the questions remain unanswered. In approximately 24% of the cases, members repeatedly ask the same questions but still receive no answers, leaving their needs unmet. For instance, one member asks about changing visa status from L1 to F1 but receives no responses. Ten days later, the same member posts a paraphrased version of the same question, yet still fails to receive any assistance in the subreddit.

**Q1** *: I’m on L1B visa which expires soon. Since I’ve been recently accepted into a Master’s program, I plan to leave the US and go back to home country to obtain F1 visa. Any advice? (March 23rd)*

**Q2** *: Is going back to my home country to change my status from L1 to F1 the best option? (April 4th)*

Another issue is the dissemination of inaccurate or irrelevant information. We frequently encounter comments deleted by moderators for containing incorrect information. Additionally, responses to certain questions often conflict with one another, leading to confusion and potential misinformation.

*“Removed as it does not address the question.”*

*“Removed for incorrect information.”*

The other example involves a question about bringing a copy of the I-20 form to a visa interview. The responses vary significantly, with some members affirming its acceptability, while others advise against it. This inconsistency creates confusion and leaves the members’ question unresolved.

**Q** *: Hello, my I-20 has arrived. Can I bring a copy of the I-20 to the visa interview?*

**Comment1** *: Yes, you can.*

**Comment2** *: No, you cannot.*

The last type of unmet information need is outdated content, where members struggle to obtain timely and relevant information. This issue becomes particularly pronounced during the COVID-19 pandemic, as rapidly changing circumstances render previous advice obsolete. For example, a member seeking information about the timeline for receiving a Social Security Number (SSN) encounters difficulties because responses do not account for delays caused by the pandemic.

**Q** *: I started my internship, but still didn’t receive my SSN yet. What should I do?*

**Comment** *: Sorry, because of COVID-19, everything has changed. Just keep waiting for HR’s response.*

**Patterns in comments.** Next, we observe a distinct pattern in comments among international students who frequently post on *r/f1visa*. Specifically, those who revisit and comment on their own questions do so with increasing frequency, indicating an ongoing search for answers or clarification. Our analysis identifies 2,037 authors who engage in this behavior, commenting on their own posts to continue or close the discussion.

The likelihood of this engagement increases with the number of questions individuals ask. While only 45% of those who post once return to comment on their own posts, this number increases as the number of posts by individual members rises. A deeper dive into the data shows a progressive increase in engagement for members asking more questions: 68% for two-time askers, 77% for three-time askers, and notably higher percentages for those asking questions even more frequently (Table 7). This trend culminates in a 92% engagement rate among those who post five times, highlighting the persistent pursuit of unmet information needs.

**Table 7:** Ratio of self-commenting to the number of posts.

Number of Posts	Self-Commenting Rate of Posts
1	0.4556
2	0.6842
3	0.7725
4	0.8876
5	0.9207

An examination of the self-comments reveals a shift in language that reflects the evolving nature of these inquiries. Comments often include expressions of gratitude, as indicated by the prominent cue “thank” among those asking a single question. For example, in one case, an author submits a single post regarding finding financial assistance to pursue an academic journey. A comment (referred to as “Member comment” below) offers both potential solutions and emotional support, stating, *“Don’t give up your dreams!”*. The author then responds to express appreciation for the advice by using the linguistic cue *“Thank you for all your descriptions.”*

**Q** *: I recently completed my undergrad but had to graduate early due to financial difficulties, or I had to drop out. I want to go to medical school but do not want further debt. I am stressed out about my financial situation but don’t want it to stop me from pursuing my education. Please help! Any and all advice would be greatly appreciated.*

**Member comment***: I understand what you’re going through—it’s tough in the U.S. I come from a well-off family in my home country, but even for us, it was hard when I moved here. My parents had to work extra and delay their retirement to support me. (...) There is a scholarship at [certain institution]. You can also check out [this website]. Also, some of my friends pursue their education in other countries like U.K., (...).* **Don’t give up on your dreams!**

**Self comment**: **Thank you for all your descriptions***. I’ve gone over them several times. It was one of the most difficult situations. (...)*

**Fig 4:**
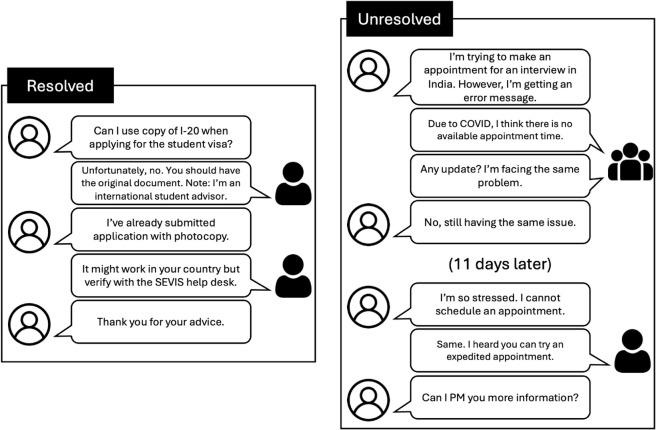
Examples of a case that is resolved with a single post (left) and a case that remains unresolved after two posts (right).

In contrast, members posting multiple questions tend to use cues such as “need” and “still.” This highlights ongoing information gaps despite previous attempts to obtain answers. For example, in Fig 4, a member states, *“No, still having the same issue”* to express a continued need for information even after receiving answers.

Therefore, our findings suggest that asking recurring questions is not merely a function of habit or community engagement but rather a manifestation of deeper, unmet information needs among international students. This is particularly evident among those who engage more frequently in the subreddit, indicating that initial responses may not have fully addressed their concerns or that new questions arise from previous answers.

Analyzing the distribution of the number of posts per author, we find that with a 95% confidence level, the upper bound is set at five posts. Authors with more than five posts are considered outliers, especially active in communicating within the subreddit. The analysis of outlier users who post five or more times reveal a dramatic shift in international students’ information-seeking behavior before and during the pandemic. In the pre-pandemic period, only 2 users contribute 79 recurring questions, primarily functioning as community moderators or advisors providing systematic guidance on F1 visa fundamentals. Their posts mostly focus on regulatory information such as I-94 documentation and OPT/CPT employment opportunities, often linking to official resources and job platforms. Their posts reflect a top-down, guidance-oriented pattern in which a small number of experienced users served as knowledge brokers for less experienced students.

During the pandemic, however, the profile of highly engaged users transforms significantly, with 107 users generating 933 recurring questions. Their recurring questions focus on visa appointments and suspensions, international travel restrictions, OPT delays, unemployment benefits, and mental well-being—topics reflecting the uncertainty and disruption of COVID-19. Moreover, these engaged users increasingly seek assistance with practical daily life matters (tax filing, banking, COVID-19 vaccine, insurance, driver’s licenses) and emotional support regarding employment stress and mental health challenges. This shift indicates that recurring posters changed from a small group of knowledgeable community guides to a broad cohort of students persistently navigating unprecedented disruptions and seeking both procedural guidance and peer support. This reflects how the pandemic’s complexities intensify international students’ information needs while simultaneously fostering a more active and mutually supportive community.

Building on this insight, further exploration into international students’ behavior on Reddit reveals another trend: those with recurring posts often revisit similar topics within a short timeframe, suggesting that their initial information needs remain unfulfilled. This recurrent posting behavior indicates a deeper unmet need for support and information that persists despite multiple attempts to obtain assistance. Our observations are illustrated in Fig 4, where we distinguish between two types of members: one who, after a single post, concludes with a word of gratitude, *“thank you,”* suggesting their question is resolved, and the other who, after expressing ongoing issues, responds with *“no, still facing the same issue,”* and reposts the same question 11 days later, indicating unresolved challenges but a glimmer of hope in finding a solution through community engagement.

## Discussion

In the context of the COVID-19 pandemic, our analysis reveals an increase in both the diversity and volume of inquiries posted by international students on Reddit. This trend aligns with findings from earlier studies on the general population, which also identify a rise in the diversity and volume of information being shared due to heightened uncertainty, anxiety, and the policy changes brought about by the pandemic [98]. This shift reflects a change in international students’ information needs, indicating an intensified and altered demand for information following the pandemic’s onset (RQ1), while certain aspects of these needs remain unmet (RQ2). Below, we discuss design and societal implications based on these research findings.

In line with previous research [9], our study confirms that international students seek legal and job-related information more frequently than academic information. As part of our findings for RQ1: Topics Related to the Information Needs of International Students, we observe that common topics, such as visas and EAD cards, persist before and during the COVID-19 pandemic, but the focus of inquiries shifts notably. Pre-pandemic topics focus on job applications and fields like data science and law, while pandemic-era discussions broaden to travel restrictions, financial struggles, working hours, and funding, reflecting greater anxiety and difficulties. Even familiar topics, like passports, shift from neutral applications to concerns about refusals, further highlighting this vulnerability.

Our findings also suggest that the similarity among recurring questions increases during the pandemic, aligning with the focus of RQ2: Recurring Unmet Information Needs. This pattern not only highlights the unmet information needs of international students but also underscores Reddit’s potential as a crucial platform for information dissemination and support. International students who post repeatedly are more likely to return to their posts to actively communicate with other international students, despite their information needs appearing to be inadequately addressed, compared to those who post on Reddit only once.

### Theoretical contributions

This study extends Wilson’s model of information-seeking behavior [40] to explain collective patterns in digital environments. Repeated postings and paraphrased questions reflect persistent information needs shaped by structural barriers such as limited institutional transparency and inconsistent official guidance. These patterns align with Wilson’s concept of intervening variables—psychological, social, and environmental factors, including anxiety, self-efficacy, visa status, and institutional constraints—that influence how individuals act on their information needs [40].

Reddit’s role as an informal peer-to-peer information source further highlights how social validation and emotional coping become central to information seeking. Under heightened uncertainty and isolation, particularly during the COVID-19 pandemic, affective drivers replace purely instrumental motivations. Users’ repeated visits to threads for updates or clarification illustrate information seeking as an iterative, adaptive process shaped by continuous feedback loops.

This study also extends Information Foraging Theory (IFT) [42–44], showing how information-seeking adapts under crisis conditions. As institutional guidance fluctuates and misinformation risks increase, users favor re-exploitation strategies—returning to familiar information patches rather than exploring new ones. Recurring posts and follow-up queries reflect a strategic recalibration in which exploration incurs higher cognitive costs, while exploitation—refining existing information—offers greater perceived benefit. These temporal dynamics reveal how crisis conditions amplify reliance on re-exploitation, intensify information-patch revisits, and make adaptive iteration central to the foraging process. In this context, online communities like Reddit function not as passive repositories but as active ecosystems where cost–benefit decisions are continuously recalculated as uncertainty unfolds.

#### Social captial theory.

This study extends social capital theory by illustrating the adaptive nature of online communities during crises. Following the onset of COVID-19, engagement in the *r/f1visa* subreddit surged. Posts increased from 534 to 7,472 and comments from 1,325 to 32,656, indicating that the forum functioned as a vital form of online social capital, enabling international students to exchange information and emotional support amid uncertainty.

Because Reddit users differ by nationality, institution, and academic background, the community primarily embodies bridging social capital [55]. Yet under crisis conditions, these weak-tie networks evolve, as shared stressors foster empathy and solidarity. The subreddit thus became a hybrid space where bridging ties ensured informational diversity while bonding elements deepened through mutual recognition and collective resilience.

This leads to a theoretical refinement: during crises, online bridging capital can generate bonding-like benefits—emotional support and collective identity—without the dense, enduring ties typical of traditional bonding capital. Such hybrid formations challenge the binary distinction between bridging and bonding social capital, showing how digital platforms enable new configurations in which structural diversity and situational solidarity coexist.

### Practical implications

The findings highlight several actionable steps that universities, policymakers, and online community moderators can take to address the unmet information needs of international students (RQ2). For universities, this includes developing proactive communication strategies, such as regular information sessions, multilingual resources, and real-time updates on policies that directly affect international students. Policymakers, particularly those involved in immigration and higher education, could improve the clarity and accessibility of official guidelines to reduce uncertainties that lead students to seek information from unofficial sources. Additionally, Subreddit moderators may also play a role by curating frequently asked questions, pinning authoritative resources, and collaborating with verified experts to ensure the accuracy of shared content.

The behavior of subreddit users indicates a strong likelihood for international students to address their information needs through multiple channels [99], which is particularly important given the time-sensitive nature of their concerns within constrained time frames. Original posters can add value to the forum by returning to share updates or outcomes, transforming unresolved posts into great references for future users. Maintaining this ecosystem could help mitigate the unmet information needs resulting from a lack of responses.

Although the top-down moderation system on Reddit, managed by human moderators, leverages sociocultural knowledge to provide nuanced perspectives [100], it can also hinder user engagement, particularly in subreddits like *r/f1visa* that emphasize credibility and quality or serve marginalized communities. For instance, moderators may remove comments left by users simply to track updates on posts of interest, as such comments can push down comments with substantive information. This highlights the need for personalized follow-up systems to maintain engagement without compromising the subreddit’s information quality. To support revisiting behavior while respecting the subreddit’s environment and moderators’ efforts, Reddit developers could implement strategies to encourage users to revisit and update their posts. For example, email reminders have been shown to increase participation in online communities [101]. Personalized alerts reminding users to share updates could further enhance both the quantity and quality of information shared on the platform.

Another type of identified unmet information need is the presence of inaccurate or irrelevant information. Reddit has become a critical source of information for international students, operating on trust comparable to that of personal networks, which are traditionally key sources for this group [102]. Due to this reliance, some moderators of subreddits enforce strict rules and manually manage posts and comments—removing inaccurate, outdated, and unrelated content from the community’s feed. While automated verification using tools like LLMs (e.g., ChatGPT) could be promising, these models are not well-suited for visa-related inquiries due to the legal complexities involved. LLMs often lack the ability to provide legally accurate advice and logical analysis, which are critical to assessing the credibility and relevance of such information [103]. Additionally, ChatGPT is trained on static datasets, rendering their knowledge outdated as of their last update. Even within their training scope, they often fail to provide consistent or current information, which can lead to the dissemination of outdated facts and, in some cases, misinformed decisions with potential legal implications [104]. Moreover, LLMs may exhibit a higher tendency for hallucinations, misinformation, stereotypical representations, and potential misuse, amplifying vulnerabilities for international students compared to their domestic counterparts [104].

To address these challenges, AI-assisted moderation tools tailored to Reddit’s needs could complement human moderators by flagging potentially inaccurate or irrelevant content, prioritizing posts with high engagement or frequent reports. This aligns with prior research suggesting the use of automated tools, such as emotion detection systems, to enhance social interaction within online communities [76]. Additionally, beyond Reddit’s existing upvote-downvote system, community-based verification mechanisms, such as badges, could be introduced. Badges, widely used in online communities, serve functions like establishing authority, group affiliation, and identity [105]. Originating from gaming and often associated with “gamification,” badges are earned through specific user behaviors and can effectively motivate community members to engage in desired activities.

This verification mechanism could also be adapted to tackle outdated information effectively. Introducing optional metadata fields for original posters and commenters—such as the year of admission, graduation, visa application or job-seeking timelines—could help readers assess the relevance and timeliness of the shared information while maintaining anonymity. To encourage the community’s active participation, moderators could implement a feature allowing users to mark posts as outdated or confirm updates when the context changes. For example, a “Verified as Current” tag could be added to information confirmed as up to date, while an “Outdated” label could be displayed for posts reviewed and deemed no longer applicable. Combined, these strategies would empower international students to navigate better and trust the subreddit while ensuring the information remains accurate and relevant over time.

### Societal implications

Beyond institutional and policy implications, this study reveals several societal implications. The evolving information needs of international students, especially at the onset of the COVID-19 pandemic, call for a more adaptive approach to information provision. Moreover, we observe unmet needs stemming from their limited knowledge and resources within the subreddit. Our analysis could contribute to policy-making through collaborative online environments [106]. By highlighting topic shifts within this forum, this study aids in designing timely coping strategies that can be leveraged by both individuals and societal stakeholders. Building on this understanding, policymakers and institutions should focus on delivering timely, accessible information that addresses students’ changing priorities and challenges, particularly in times of crisis. For example, travel and visa acquisition emerge as critical and recurring concerns for international students during the pandemic in our study. Restrictive travel and visa policies disrupt academic plans, hinder students’ ability to move between their home countries and the U.S., and negatively impact their mental health and well-being [107]. To address these challenges, cooperation between universities, the U.S., and the home countries of international students may be necessary to establish effective dissemination channels for timely information access. Similarly, despite the financial support available to native-born students, international students are not eligible for financial aid [108]. Given the worldwide negative financial impact, international students may struggle to sustain their studies in the U.S. Consequently, this vulnerable population would benefit from increased financial aid and support, including comprehensive crisis management planning.

In addition to these policy-level responses, the prevalence of unmet information needs among international students, as evidenced by their online interactions, highlights the critical role of community-driven platforms in providing support and guidance, in line with previous study [69,109]. Higher education institutions, particularly international student affairs offices, should leverage Reddit to disseminate accurate information and resources. Moreover, higher education institutions could enhance their support by developing targeted FAQs, webinars, and other resources that address the specific concerns of international students, thereby implementing more tailored communication strategies.

Beyond these immediate practical implications, our findings illuminate broader societal dynamics that shape international students’ experiences. In particular, two complementary lenses reveal the deeper structural and psychological dimensions of information seeking in crisis contexts. This study aligns with the framework of cross-cultural adaptation, which views adjustment to a new sociocultural environment as a dynamic process of coping, learning, and growth. According to the stress–adaptation–growth dynamic, individuals entering unfamiliar settings experience psychological and behavioral stress that triggers adaptive responses aimed at restoring balance [110]. International students’ use of Reddit reflects these adaptive mechanisms, as they seek information, share experiences, and provide mutual support to navigate institutional systems and cultural expectations. Reddit also serves as a venue for maintaining and creating latent connections that foster belonging and access to informal knowledge networks [111]. These adaptive practices become especially salient during the COVID-19 pandemic, when institutional communication is delayed or fragmented. Posts reveal patterns of collective sense-making and risk interpretation, as users collaboratively clarified policies, exchanged timely updates, and offered emotional reassurance, reflecting core functions identified in crisis communication frameworks that emphasize information sharing, meaning construction, and coping under uncertainty [112,113].

Complementing this adaptive perspective, we advance the understanding of digital inequality by moving beyond first-level (access) and second-level (skill-based) divides to examine third-level, outcome-based disparities. Although international students are digitally connected and competent Reddit users, their institutional information channels often fail to meet their needs, resulting in unequal access to credible and timely information. This reflects the third-level digital divide, which concerns inequalities in the outcomes derived from internet use rather than access itself [114]. These disparities show that digital inequality persists through differences in individuals’ capacity to mobilize online platforms for coping, learning, and adaptation under structural and situational uncertainty [115]. Consistent with the view that digital inequality intersects with race, class, and gender and is shaped by offline circumstances [116], this study illustrates how international students use Reddit to compensate for institutional and informational gaps. While access disparities are minimal, variations in effective use linked to social position, system familiarity, and peer networks can produce uneven informational and emotional outcomes. By analyzing these dynamics across large-scale, user-generated data, this study provides empirical, at-scale evidence of how outcome-level disparities manifest in online peer information environments.

## Limitations and suggestions for future work

This study has several limitations that should be acknowledged. First, the exclusive reliance on Reddit may introduce bias, as the platform’s user base skews toward certain demographic groups, particularly younger, male, and more tech-savvy individuals [88]. This limits the generalizability of the findings to broader populations. Furthermore, due to the nature of big data, the analysis does not allow for the identification of individual characteristics of international students, which constrains a more nuanced understanding of their information-seeking behaviors. Second, the analysis does not allow for the identification of individual characteristics of international students, which constrains a more nuanced understanding of their information-seeking behaviors. For instance, international students from Asian countries have faced harsh stigma and discrimination during the COVID-19 pandemic [117], and research indicates that female international students are more mentally vulnerable than their male counterparts [118]. Consequently, the information that international students seek may vary according to race and other sociodemographic characteristics. Moreover, prior studies suggest differences may exist in the information needs and information-seeking behaviors between undergraduate and graduate students [119]. However, our study does not distinguish these groups to analyze and compare their information-seeking behaviors and unmet needs. Another limitation of our study lies in its inability to fully encompass the diversity derived from non-WEIRD populations. While several works highlight the importance of incorporating non-WEIRD perspectives to achieve a more comprehensive understanding and thus better inform community design [120,121], this study focuses solely on international students. Although international students in our study may provide a partial representation of cultural diversity, they do not fully capture the wide range of socioeconomic contexts, linguistic backgrounds, and cultural norms that exist in non-WEIRD communities. This suggests that future studies should extend beyond a single online platform to incorporate a more diverse set of populations and environments, ultimately leading to more robust insights into community design for online information-seeking.

Building on these findings, we offer several recommendations for future research. It would be beneficial to explore whether consistent patterns of information-seeking behavior arise from unmet needs across various sources, including traditional outlets and other online-based platforms, such as Facebook and Quora. Also, a hybrid approach, incorporating AI-driven information seeking through Large Language Models (LLMs), could serve as an additional resource for international students’ information seeking.

While this study demonstrates that Reddit enables peer-based information exchange among international students, it also functions within an infodemic environment where false and factual information spread at similar speed, blurring perceptions of credibility and trust [122]. This dual dynamic complicates Reddit’s role as an informal guidance source, particularly when misinformation intersects with students’ uncertainty and vulnerability. Although social media frameworks emphasize connection and coordination during crises [112], misinformation simultaneously undermines trust and collective sensemaking in user-driven spaces. Moreover, digital inequalities are cumulative and sequential, meaning that pre-existing disparities in language, access, and digital literacy compound over time, heightening vulnerability to unreliable information [114,122]. Addressing these challenges requires moving beyond single-axis analyses of digital exclusion and developing inclusive strategies that strengthen critical evaluation skills and digital resilience. Future research should employ cross-platform and longitudinal approaches to examine how misinformation circulates within online student communities and how it shapes information-seeking behavior, trust formation, and perceived social support over time.

While this study examines Reddit as a case of online social capital that primarily exhibits bridging characteristics, where international students from diverse national, institutional, and disciplinary backgrounds exchange information, it also reveals how crisis conditions cultivate bonding-like qualities within structurally bridging networks. Future research can further explore this phenomenon by comparing international student communities across platforms or contexts that differ in their baseline levels of bonding versus bridging capital. For instance, studies may examine more homogeneous communities organized around specific national origins or shared racial and ethnic identities to assess whether tighter bonding networks better resolve recurring information needs or provide distinct forms of social support (e.g., greater emotional validation but less informational diversity). Additionally, longitudinal analyses tracking how the bonding–bridging balance shifts across crisis phases, from acute disruption to adaptation to recovery, can illuminate the temporal dynamics of online social capital formation. Such comparative research deepens understanding of how network structures influence not only the type and quality of information exchanged but also the persistence of unmet needs and the psychological functions that online communities serve when instrumental problems remain unresolved.

Finally, although this study focuses on the challenges international students faced during the COVID-19 pandemic, where quickly evolving information creates gaps that existing resources could not fill, other external shocks such as financial crises, natural disasters (e.g., tornadoes, wildfires, earthquakes), or political instability could similarly disrupt access to necessary information. In these contexts, online communities like Reddit may provide a space for students to connect with peers, share information, and access diverse perspectives in real time.

Future research could explore how international students use Reddit during different crises to meet their informational needs and whether these needs are addressed differently depending on the nature of the external shock. Specifically, studies could examine the types of information students seek, how they leverage these platforms to build social networks, and the role of Reddit in providing emotional support and practical resources. By investigating these dynamics, future studies could gain a deeper understanding of how online communities function as essential support systems for international students in times of uncertainty and disruption.

Beyond the primary focus on information-seeking patterns, this study also identified misinformation as a recurring barrier within Reddit discussions, particularly concerning visa regulations, work authorization, and institutional procedures. Although not systematically analyzed, these instances illustrate how peer-based platforms can simultaneously reduce uncertainty and perpetuate inaccuracies in high-stakes contexts. Future research should more explicitly examine the prevalence and consequences of misinformation and explore mechanisms to enhance information verification and reliability in online peer communities.

## Conclusion

This study examines how the information-seeking behaviors of international students on the subreddit *r/f1visa* evolved before and during the COVID-19 pandemic, revealing significant shifts in topics reflecting changing challenges. Faced with unmet information needs, such as unanswered questions or incomplete answers, students demonstrate active engagement by posting follow-up comments, revisiting unanswered posts to seek clarity. These behaviors highlight the critical role of online communities like Reddit in supporting international students as they navigate uncertain situations. Enhancing the reliability and timeliness of information on such platforms can better address these evolving needs, while future research could explore intergrating AI tools to further optimizer peer-driven support systems.

## Supporting information

S1 Fig Word clouds of comments. The left word cloud analyzes comments on posts created by members who post only one post. The right word cloud analyzes comments on posts by authors who have posted more than five posts.
(TIFF)
