# Supplementary figures and images for: “I Still Need Your Help”: Online information seeking behavior of International Students in the United States on Reddit
Source: PLoS One. 2026 Feb 9;21(2):e0341314. doi: 10.1371/journal.pone.0341314 (PMC12885376; doi:10.1371/journal.pone.0341314)

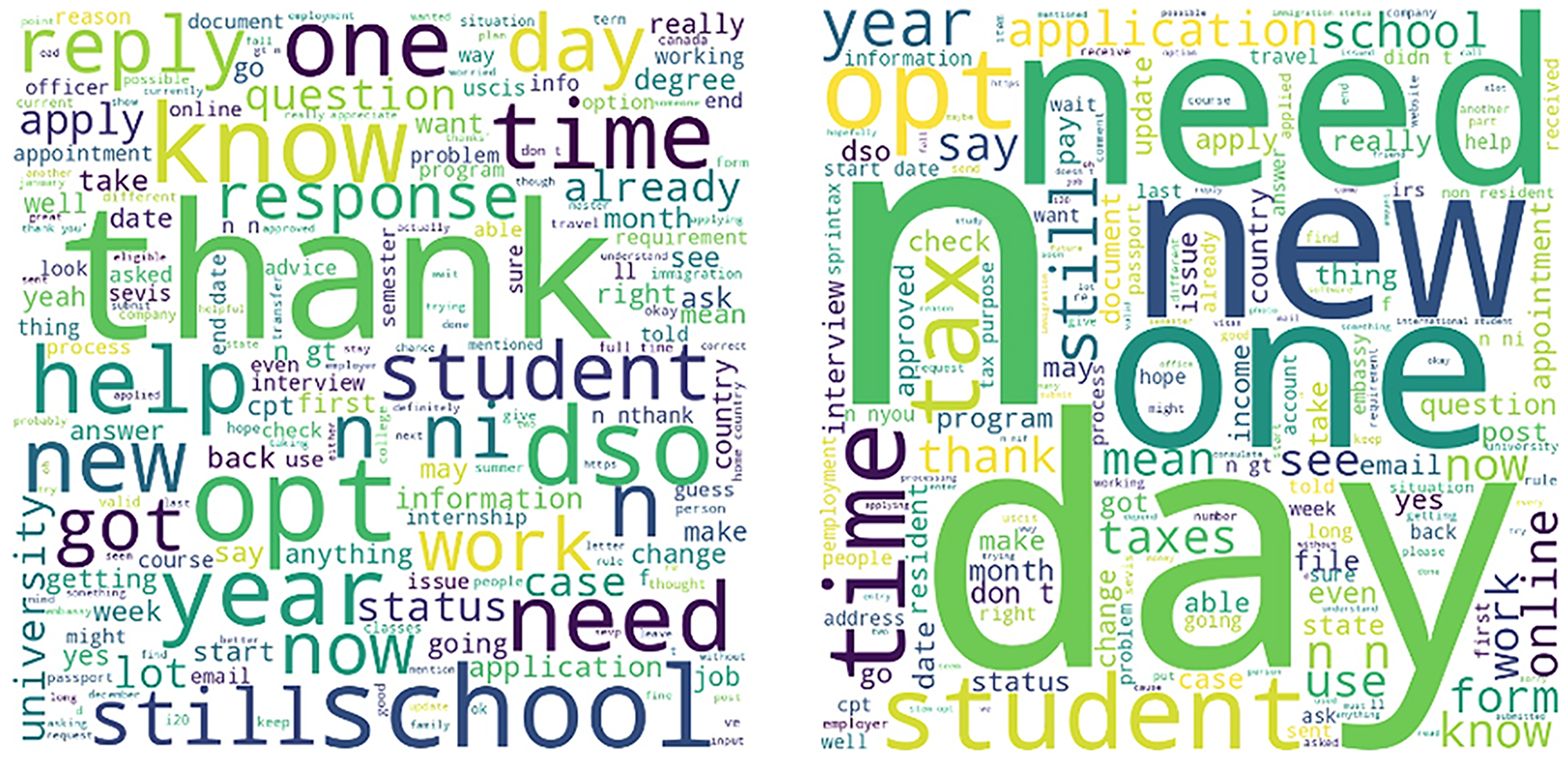

Supplement: S1 Fig — The left word cloud analyzes comments on posts created by members who post only one post. The right word cloud analyzes comments on posts by authors who have posted more than five posts. (TIFF) [file pone.0341314.s001.tif]
